# A qualitative study to understand people’s experiences of living with Charcot neuroarthropathy

**DOI:** 10.1111/dme.14784

**Published:** 2022-01-14

**Authors:** Catherine Gooday, Wendy Hardeman, Frances Game, Jim Woodburn, Fiona Poland

**Affiliations:** ^1^ School of Health Sciences, Faculty of Medicine and Health Science University of East Anglia Norwich UK; ^2^ School of Health Sciences – Behavioural and Implementation Science Group, Faculty of Medicine and Health Sciences University of East Anglia Norwich UK; ^3^ Department of Diabetes and Endocrinology University Hospitals of Derby and Burton NHS Foundation Trust Derby UK; ^4^ School of Health Sciences and Social Work Griffith University Southport Australia; ^5^ School of Health Sciences ‐ Institute for Volunteering Research, Faculty of Medicine and Health Science University of East Anglia Norwich UK

**Keywords:** Charcot neuroarthropathy, diabetes complications, guilt, pain, qualitative research, social participation, social support

## Abstract

**Aims:**

Charcot neuroarthropathy (CN) is a complication of neuropathy, in people with diabetes. Treatment requires the prolonged wearing of an offloading device, which can be challenging. The importance of understanding people's perspectives for promoting their engagement in self management is well known. However, no such studies have been done in CN. This qualitative study aimed to understand people's experiences of CN.

**Methods:**

Semi‐structured interviews with a purposive sample of 14 participants with CN, recruited from a randomised controlled trial. We gathered opinions, thoughts and the meanings participants attributed to their experiences of CN and its physical, socio‐economic and physiological effects and how this affected their families and relationships. We analysed the interviews using Inductive Thematic Analysis.

**Results:**

Four analytic themes were identified: (1) *‘*Trapped at home isolated and missing social life and daily life routines’; (2) ‘Disruption to people's roles, responsibilities, relationships and mobility, which people adapted to try and address and manage’; (3) ‘Pain which participants related to the direct or indirect consequences of wearing the cast or boot’; and (4) ‘Blame for developing CN, attributed to themselves and healthcare professionals’. Participants described guilt about needing more support, expressing frustration, low mood and low self‐esteem.

**Conclusion:**

This study highlights experiential aspects of the previously unrecognised burden of CN. Its physical, social and emotional impacts on participants and their families are substantial and sustained. There is a need to raise clinical awareness of CN and its wider effects.

**Trial registration:**

ISRCTN74101606. Registered on 6 November 2017, http://www.isrctn.com/ISRCTN74101606?q=CADom&filters=&sort=&offset=1&totalResults=1&page=1&pageSize=10&searchType=basic‐search

**Novelty Statement**

- This study has shown that receiving treatment for Charcot neuroarthropathy has physical, socio‐economic and psychological consequences, which extend beyond the burden of wearing an offloading device.
- Participants were frustrated about the impact of living with Charcot neuroarthropathy and experienced low mood and low self‐esteem. The physical and emotional effects of living with Charcot neuroarthropathy on participants and their families were substantial and sustained.
- To limit the negative consequences of living with Charcot neuroarthropathy, there is a need to increase awareness of Charcot neuroarthropathy. Health and social care professionals should adopt a more holistic approach to supporting individuals with Charcot neuroarthropathy.

## 1 BACKGROUND

Living with any long‐term condition can affect people's lives, and change people's roles and responsibilities, financial situation and housing needs.^1^ These changes can affect the individual, their families and their relationships and for all involved they can be difficult to accept and adapt to. It is known that living with diabetes has a negative effect on people's experiences and their emotional well‐being, with higher levels of depression and other mental health problems than the general population.^2^ Having a diagnosis of diabetes also reduces people's health‐related quality of life.^3^ In type 2 diabetes, developing diabetes‐related complications has been associated with further‐reduced health‐related quality of life.^2^

Foot and ankle complications including, ulceration, amputation and Charcot neuroarthropathy (CN) represent a major socio‐economic challenge because of the affect they have on people's physical and psychological function.^4^ Foot complications place a financial burden on people with diabetes,^5^ their families and the healthcare system.^6^ To date, qualitative research around diabetic foot complications has focused on people's experiences of preventing and managing foot ulceration and amputation. A qualitative meta‐synthesis of 42 papers on the patient's perceptions and experiences of diabetic foot care found that foot ulceration had significant and long‐term physical, socio‐economic, psychological and interpersonal consequences.^4^

CN is a complication of diabetes associated with neuropathy which primarily affects the foot and ankle. It is a progressive condition that affects the bones, joints and soft tissues. There is uncontrolled inflammation and bones become osteopaenic which can lead to fractures, joint dislocation, deformity and ulceration. Treatment aims to stop the inflammatory process, relieve pain and maintain foot architecture by wearing an offloading device, usually a non‐removable below knee cast or walker boot.^7^ Studies from the UK show a median time to remission of between 9 and 12 months.^8^, ^9^ However, international studies report considerably shorter time to remission, in the United States of 3–5 months,^10^, ^11^ in Brazil and Germany 3–12 months and 3–6 months, respectively.^12^, ^13^ We do not fully understand the impact that wearing an offloading device for this length of time can have on peoples’ physical, psychological and social comfort.^14^

We do not know whether the findings from research into experiences of people with foot ulceration are relevant to people with CN. The evidence about the effects on people living with CN comes from quantitative findings about how people experience changes in anxiety, depression and quality of life, and not about peoples’ lived experiences.

The National Institute for Health and Care Excellence NICE (2015) guidelines ‘Diabetic foot problems: prevention and management’ recommended more in‐depth research into the health‐related quality of life of people with CN.^15^ A more detailed and nuanced understanding of how people live with CN could encourage more effective and constructive relationships between people with CN and healthcare and social care professionals. Gaining an understanding of the physical and psychosocial experiences of people with diabetes and CN could help develop interventions to improve experiences of people receiving treatment for CN and so reduce personal, healthcare and social care costs.

To address this research gap, this study sought to capture the participants’ experiences of living with CN.

## 2 AIM AND OBJECTIVES

In this qualitative study, we aimed to further the understanding of people's experiences of CN. The objectives were to explore:

- The perceived effect of CN on day‐to‐day functional activities.
- The effect of living with CN on social participation.
- How receiving treatment for CN may affect people's relationships with family, friends and colleagues.
- The effect of these experiences on people's sense of self and self‐worth.

## 3 PARTICIPANTS AND METHODS

We recruited a sample of participants from people with confirmed CN who took part in a feasibility trial on the use of serial MRI in disease monitoring. The inclusion and exclusion criteria for the feasibility trial have previously been reported.^16^ The interviews were carried out in secondary care clinics between August 2019 and January 2020.

A sample size of 10–14 was set, based on recommendations for strategic and practical reasons of ensuring adequate information from the widest range of people.^17^ Five participant characteristics were chosen to purposively inform the sampling framework to maximise variation: sex, age, history of previous foot complications, duration of treatment for the current episode of CN and employment status. These characteristics were selected to identify shared patterns that cross cases and ensure that unique or diverse experiences of CN were captured.

Face‐to‐face semi‐structured interview data were collected using a topic guide (Data S1). The knowledge and experience of the clinical members of the research team, and the findings from the literature review informed the initial framework for the topic guide. The topic guide was then refined following feedback from patient and public representatives. We sought to collect participants’ self‐accounts of their opinions, thoughts, feelings and to identify meanings that they attribute to different CN‐related areas of experience.

All participants provided written consent to take part in the feasibility trial and were re‐consented by a member of the research team prior to participating in the qualitative interviews.

The interviews were recorded and then transcribed. We used Inductive Thematic Analysis and the six‐step model to analyse the data.^18^ In this process, the data are subjected to a rigorous analysis over six steps: (1) familiarisation gaining familiarity with the data, (2) generating initial codes, (3) searching for themes across codes, (4) reviewing themes, (5) defining and naming themes, and then (6) sharing the findings with healthcare and social care professionals, policymakers and people with diabetes. One researcher (CG) read all the transcribed interviews to record emerging ideas, then coded the transcriptions line‐by‐line supported by NVivo12. The initial coding framework was refined by a second researcher (FP) and cross‐checked against a small sample of transcripts. The coded data were then abductively thematically analysed, identifying key categories and themes (Data S2). To enhance the credibility of the analysis, we produced a newsletter capturing the key themes, with illustrative examples as an engaging means to ask participants, how far these themes capture their own experiences of living with CN.

## 4 RESULTS

In all, 42 of the 43 participants in the feasibility study agreed to be contacted about the qualitative study. We interviewed 14 participants whose characteristics are summarised in Table 1. Participants wore a mixture of non‐removable and removable below knee casts/boot. Participants were selected in sequence to ensure they would meet the sampling framework criteria and achieved a maximum varying sample. No participants were excluded from the study. We identified four key themes:

- ‘Trapped at home isolated and missing social life and daily life routines’.
- ‘Disruption to people's roles, responsibilities, relationships and mobility, which people adapted to try and address and manage’.
- 'Pain which participants related to the direct or indirect consequences of wearing the cast or boot'.
- 'Blame for developing CN attributed to themselves and healthcare professionals'.

**TABLE 1:** Participant characteristics

Baseline participant characteristics	*n* = 14
Study details	
Duration of participation in study median [25^th^–75^th^ IQR]	161 [103.5–241.75]
Intervention arm *n* [%]	8 [57%]
Sociodemographic	
Men *n* [%]	8 [57%]
Age (years) mean ±SD	61 ± 9.1
Highest education *n* [%]	
Stayed in school until 16	6 [43%]
Stayed in education until 18	3 [21%]
Vocational/occupational, training/qualification	4 [29%]
Degree	1 [7%]
Non‐removable knee‐high offloading device (cast or boot) *n* [%]	6 [43%]
Working at diagnosis *n* [%]	6 [43%]
Previous minor amputation *n* [%]	4 [29%]
Previous CN *n* [%]	3 [21%]

### 4.1 Trapped at home isolated and missing social life and daily life routines

The theme ‘trapped at home isolated and missing social life and daily life routines’ was voiced by all the participants. While everyone interviewed expressed these feelings, across the data there were differing nuances, often reflecting individuals’ different circumstances before diagnosis.

Expressions of isolation were mainly associated with ‘physical isolation’ where the offloading device restricted participants’ social interactions. Social isolation resulted from a combination of factors: disability caused by wearing the cast making it more difficult to go out, distance as people could not easily access public transport to visit family and friends who did not live locally, and for a few participants a perceived social stigma about wearing the offloading device. They reported on how this affected casual social interactions, such as meeting and talking to people when out shopping and during formal or planned interactions such as going to work, meeting family, friends or attending clubs.

*I can’t do nothing; can’t obviously… can’t do stairs or anything. Um, I’ve had to finish my job because it involved all walking. P3 female, aged 50–60*

However, one participant reported experiencing both ‘physical and emotional isolation’; they were unable to go out to meet people and their relationship with their partner had broken down as a direct result of their being unable to do the things they used to do. They experienced rejection and being ostracised within their own home. Being isolated led participants to report feelings of low mood. While not all relationships had broken down, participants with spouses, partners and children all described how restrictions in their own mobility also affected their relations with others in various ways.

*Oh, here’s a thing – my wife’s on at me because it’s limited her social life. The limitations and the future. Um, it’s alright saying well, its four months out of your life, but you try telling my wife that. P9 male, aged 60–70*

Realising these limitations contributed to participants’ feelings of guilt and being a burden, sometimes leading to friction in relationships, and to further stress and anxiety for the individuals involved. Participants described how not only did their partners and spouses provide physical support, but also provided emotional support, without which they would not have been able to cope.

*It’s just horrible. I’m lucky I had a good one at the side of me, otherwise… [whispers] – I don’t know what I’d have done.” P5 male, aged 60–70*

Important differences in the experience of participants who had paid employment were associated with whether participants were able to continue working while wearing the offloading device, and how they perceived their current and long‐term job security. Participant reported that they missed work and the purpose it gave to the day. They also discussed how work was important for social interaction with colleagues and not being at work, contributed to them feeling isolated.

*I miss work*. *I don’t miss the job; I miss the colleagues. So, as I say, it’s not so much the place, it’s the people isn’t it. P3 female, aged 50–60*

For those participants who were in paid work before but not after their diagnosis this raised financial implications, which contributed to feelings of stress and anxiety.

*I’m not earning any sick pay and I’ve got a financial… it’s put me in a serious financial situation. It’s caused a lot of stress, sleepless nights, um…not eating. P6 male, aged 60–71*

Participants described trying to find a balance between following the advice from healthcare professionals while managing the impact of living on a reduced income. People explained how they spend less when they did not go to work, not using as much petrol, and not buying newspapers, coffees and lunches but overall, the main expenses of mortgages, rent and household bills did not change. Participants also had long‐term concerns over whether they would ever be able to return to the type of work they did before their diagnosis.

Participants identified the things they could no longer do while wearing the offloading device, and then discussed how not being able to do the activities they had previously enjoyed made them feel bored and contributed to feelings of low mood.

*Some days, an hour feels like a day. It's just the monotony of being within these four walls. You feel like they're closing in. I’ve gotta get out of here. P6 male, aged 60–71*

Participants went on to discuss how they had adapted and changed the things they used to do to try and fill the time and combat these feelings of low mood, frustration, monotony and boredom. A common activity which participants described replacing work or other leisure activities with was watching the television. Participants did not consider watching the television as a good substitute for the activities they have previously enjoyed, it just filled the time.

### 4.2 Charcot neuroarthropathy disrupts people's roles, responsibilities, relationships and mobility. People adapted to try and address and manage’

Thoughts and feelings around ‘disruption and adaptation’ appeared to play a pivotal role for participants living with CN and was a powerful theme common to all these participants. Participants reported many challenges while wearing the offloading device and ways in which they had adapted to overcome these. They discussed the frustrations that wearing the offloading device had caused them and how this had sometimes negatively affected their mood. The participants reported that their mobility inside and outside the home, family relationships and caring responsibilities had all changed extensively.

*I’ve gone from being very outgoing to just being at home; I don’t do nothing; I don’t get around or anything. P3 female, aged 50–60*

Often the participants self‐managed a range of underlying health conditions, some but not all related to their diabetes. Participants’ health before the diagnosis of CN influenced what level of disruption and the range of adaptations that they needed to make. Nearly all the participants had made adjustments to cope with immediate restrictions caused by while wearing the offloading device and in anticipation of future foot problems. Participants reported that they could no longer do basic household jobs such as hoovering. These types of tasks had been taken over by their partners or others in their households.

*Well, sort of housework type thing. I can’t do hoovering and that, which was my sort of duty but I don’t do that. [Laughs] I don’t shopping anymore, I get that delivered by a company P1 male, 60–70*.

Many participants had decided to make adaptations to their house to help them manage while wearing their cast or boot, and to make life easier in the future should they have further foot problems. They commented that the adaptations had made their home safer for them, made daily tasks easier to carry out and ensured they maintained their independence. Participants described purchasing anti‐slip mats for bathrooms, buying grabbers to pick up things from the floor, a reclining chair, having bathrooms adapted and fitting a stair lift. One person was in the process of a major house renovation to make their home more accessible for wheelchairs. Participants thoughts about their health in general and foot health were the main factors that influenced the level of adaptations they made.

They also described the importance of friends in helping them maintain their independence, and that they would not have managed without their support. Participants used sticks and crutches to improve stability while wearing the cast or boot, but it made relatively simple tasks such as carrying a drink or saucepans when cooking difficult. While participants expressed gratitude for help from family and friends, they also resented being more reliant on others to help and would have preferred to be able to manage on their own and so maintain their independence.

Wearing the offloading device appeared to reduce peoples’ stability in standing and walking and to increase falls risks. Participants talked about how they addressed this risk while trying to minimise it by using walking aids, wheelchairs and mobility scooters.

*I fell at home and then I fell outside accident and emergency … I know it’s got to be on, and I know it’s on for a good reason, but it just alters your life completely. P3 female, aged 50–60*

Many participants had caring responsibilities for relatives and found, that their ability to fulfil their role as a career was now reduced, which caused additional stress. Participants described how the dynamics of family relationships had altered, with some participants reporting how roles within the household had changed with husbands and/or children now taking over the housework. Some struggled and with guilt about not being able to do their fair share of the household chores. Some participants faced conflict in their relationships with spouses or partners over the change in roles and responsibilities and having less money. However, on the whole, the majority of participants described how supportive friends and family had been and how they could not have managed physical or emotionally without this help and support.

### 4.3 Pain which participants related to the direct or indirect consequences of wearing the cast or boot

Pain which participants related to the direct or indirect consequences of wearing the cast or boot was a powerful theme that emerged during the interviews and was reported by 13 of the 14 participants. Some participants commented that their current pain medication did not adequately relieve their symptoms and sought to discuss this with their healthcare team. The participants interviewed wore a mixture of devices: non‐removable and removable casts and below knee walkers. Participants had mixed opinions about which device they thought was more comfortable. Regardless of the type of device most of the participants attributed the pain they were experiencing to the offloading device being worn to protect the foot, rather than to the CN itself.

*hips hurt while I’m walking. Knees hurt when I’m walking, when they didn’t before. P1 male, aged 60–70*

Some participants reported that cast or boot intensified their nerve pain, they experience in their foot and leg. Participants acknowledged that it was important to wear the device but explained that they nonetheless wanted more support and advice from healthcare professionals on things that they could do themselves to minimise and manage the pain.

### 4.4 Participants attribute blame for developing CN on themselves and healthcare professionals

Participants thought that more understanding and awareness of CN by both healthcare professionals and people living with diabetes was important. This would improve recognition of the signs and symptoms as well as ensure prompt treatment and improve outcomes. In some cases, participants thought that their own actions or inactions had contributed to them developing CN.

*I wasn’t as strict with me insulin and things like that. As I should have been. I know what I’ve done and yeah; suffering now. I’ll lecture anyone now if they tell me that they don’t do it themselves. P14 female, aged 50–60*

Participants talked about how a lot of information was given to them by healthcare professionals when they are diagnosed with diabetes which was difficult to absorb and remember. Several participants suggested that if people with diabetes were more aware of the importance of looking after themselves and their diabetes, they may be less likely to develop further problems. Participants who thought that their diagnosis of CN was initially misdiagnosed by non‐specialist healthcare professionals reported feelings of anger and resentment.

## 5 DISCUSSION

This study identifies the previously unrecognised, distinctive and onerous aspects and life implications of the burden of CN experience. Receiving a diagnosis of CN, often without warning, frequently resulted in denial, shock, fear, anger and resentment. Analysing the semi‐structured interview data produced four themes. The first theme, ‘trapped at home isolated and missing social life and daily life routines’, highlighted the effects of social isolation whereby participants experienced resting the foot and wearing the cast/boot as restricting their interactions with others. The second theme, ‘disruption to people's roles, responsibilities, relationships, and mobility, which people adapted to try and address and manage’, focused on how participants reported being less mobile and more unsteady, which affected their ability to do household chores, shopping and care for others. The third theme was ‘pain which participants related to the direct or indirect consequences of wearing the cast or boot: participants attributed the pain to wearing the offloading device rather than the CN. The final theme ‘blame for developing CN attributed to themselves and healthcare professionals’ which participants attributed to their own actions or inactions and/or healthcare professionals missing the diagnosis.

Other studies which explored the experiences of people with diabetic foot ulceration showed that the restrictions of resting, wearing an offloading device and pain can leave people socially isolated.^19^, ^20^, ^21^, ^22^ Our participants described a disconnection from their social networks related to work, family or leisure.

Our study has provided deeper insights and context to the quantitative research which shows that CN decreases participants’ physical ability to perform tasks, such as shopping, cleaning and gardening.^23^, ^24^ Our results are consistent with the overall theme described for people with diabetic foot ulceration as a ‘lifetime of behavioural change’, with a life of fear, restrictions and pain^21^, ^22^ and social, psychological physical and economic impacts.^20^ There is a need to work with patients to balance the need to rest and offload the foot against the substantial physical limitations and emotional stresses.

Being isolated, managing and adapting to the disruption, caused by wearing the offloading device profoundly affected participants’ well‐being. Our participants described that healthcare professionals focus on the physical (e.g. offloading device) and medical issues (e.g., diabetes) associated with the CN, while attending less to emotional impact. In other studies, people with diabetic foot ulceration and amputation have reported their need for additional psychological support.^22^ Our study shows that there is still some way to go to meet this need.

The general advice from healthcare and social care professionals about overall good health and diabetes management is to be physically active and maintain a healthy weight. When people are diagnosed with CN, they are advised to be less physically active, rest the foot and wear offloading devices which further restrict their mobility. For participants in this study, these recommendations and physical limitations fostered their own concerns for their overall health and was reflected in their emotional distress.

This study has shown consistency between the thoughts and views expressed by participants on impacts on family members and those which were reported by the family members themselves in other studies. These include limitations to social activities, tensions within relationships,^20^ impaired mobility, frequent hospital visits and fear of amputation^25^

This study shows that pain has a substantial role in influencing participants overall experience of living with CN. Pain associated with wearing the offloading device was often felt in the more proximal joint sites of the knee, hip and back rather than the foot itself. This confirms the findings from other studies where participants experienced pain when using such devices.^20^, ^21^, ^26^, ^27^ Clinical teams need be more aware of and responsive to such pain experiences to ensure that they identify patients who are experiencing pain and where relevant, can then work with participants to look for solutions to effectively manage this pain, which, in turn, would reduce one of the triggers for emotional distress.

Individuals’ experiences of blame was the final theme to emerge from analysis. As in studies among participants with foot ulceration or amputation, participants reported that their actions and inactions as regards taking care of their feet: by not inspecting their feet regularly, not wearing their prescribed footwear or seeking help immediately they noticed a problem, and self management of their diabetes had directly led to or slowed down their recovery from foot complications.^28^, ^29^, ^30^ In many instances, participants engaged in what they regarded at the time as reasonable risk‐taking, trying to achieve a balance between quality of life and treatment compliance. Healthcare professionals may risk labelling participants as ‘non‐compliant’ if they do not understand the everyday difficulties people face while wearing an offloading device for several months.

In this study, some participants blamed healthcare professionals for missing the diagnosis of CN with resulting anger and frustration. The participants’ experiences are consistent with retrospective case series reports showing missed or delayed diagnosed of CN,^31^, ^32^ leading to worse outcomes. Our study is also consistent with studies from the United States and Ireland where participants blamed healthcare professionals and healthcare systems for causing foot problems to develop and for delays in receiving treatment for foot complications.^28^, ^30^, ^33^ Despite national, and international guidelines for the assessment and management of diabetic foot complications including CN, this study and other studies have found that people are still not being referred on to specialist services soon enough. This results in worse outcomes for people and increased cost to the healthcare providers.

### 5.1 STRENGTHS AND LIMITATIONS

The strength of this study is that the sample reflected the known typical characteristics of people who develop CN. However, the role of ethnicity, social and cultural differences was not specifically explored in this study and omitting this may affect issues around generalisation. Although the interviews were carried out when participants still received treatment for CN, the time since initial diagnosis was up to 6 months which could have introduced some recall bias. The interviews only captured the stories participants shared on that day. A longitudinal qualitative study may provide further insight into how peoples’ experiences change over time.

#### 5.1.1 Implications for health and social care professionals and policymakers

The overarching recommendations arising from this study are to increase awareness of CN among healthcare and social care professionals and people with diabetes. Professionals need to adopt a more holistic approach to support individuals living with CN. Healthcare professionals need to develop a therapeutic alliance with people with CN and understand the reasons behind individuals’ motivations and choices.

First, multidisciplinary diabetic foot teams should be expanded to include professionals with skills to support the profound emotional effect of CN on well‐being. Alongside this, there is an opportunity to upskill existing multidisciplinary team members to support people in a holistic way. Standard measures of depression and/or anxiety could be incorporated into clinical assessment to identify people who would benefit from support or referral to physiological services. Second, healthcare professionals need to work with people to find solutions to manage their pain. Third, multidisciplinary foot teams need to develop more formal links with social care professionals and voluntary organisations, to help participants access additional financial and non‐financial support. Fourth, we recommend improved links with physiotherapy departments to provide strategies on how to minimise the pain experienced when walking with offloading devices and make use of home or telehealth physical activity programmes already developed for people with other long‐term conditions. Finally, and importantly, there is a need to expand the role of people with CN who are experts by experience in service re‐design, thus improving their overall experience and care provided.

#### 5.1.2 Recommendations for research

The study findings highlight the need for research to better understand the reasons behind the concept of reasonable risk‐taking, balancing treatment adherence with quality of life. There is a need to develop strategies that move beyond education and actively support people to self manage their diabetes and foot complications, using behaviour change techniques such as goal setting and review, self monitoring, and habit formation.

## 6 CONCLUSION

Overall participants expressed frustration, experiencing low mood and low self‐esteem. These physical and emotional effects of CN on participants, their families and relationships were substantial and sustained. Living with CN has ramifications that extend beyond the physical limitations imposed directly by wearing the offloading device. There are further physical, socio‐economic and psychological consequences people prioritise if they are to manage their lives and their health. People with CN need to be able to access a wider range of support beyond their clinical team, to include psychological and social care services.

## ETHICAL APPROVAL AND CONSENT TO PARTICIPATE

The trial has been reviewed by East Midlands—Derby Research Ethics Committee, 04/10/2017, ref: 17/EM/0288, and conforms to the Helsinki Declaration (revised 2013). All participants provided written consent to take part in the feasibility trial and were re‐consented by a member of the research team prior to participating in the qualitative interviews.

## CONFLICT OF INTEREST

The investigators have no financial or other competing interests that impact on their responsibilities towards the scientific value or potential publishing activities associated with this study.

## AUTHOR CONTRIBUTIONS

CG is the NIHR Clinical Doctoral Fellow and Chief Investigator. CG and FG developed the initial idea for the study. CG, WH and FP designed the study. CG conducted the interviews. CG and FP completed the analysis. CG drafted the manuscript. All authors contributed to the manuscript for important intellectual content. All authors read, amended and approved the final manuscript.

## Supporting information

Data S1

Data S2
