# Supplementary material for: A qualitative study to understand people’s experiences of living with Charcot neuroarthropathy
Source: Diabet Med. 2022 Jan 14;39(6):e14784. doi: 10.1111/dme.14784 (PMC9305882; doi:10.1111/dme.14784)
Supplement: Supplementary file 1 — Data S1 [file DME-39-0-s002.docx]

Supplementary material 1 - Topic guide

A qualitative study to understand people’s experiences of living with Charcot neuroarthropathy

- Can I ask is Charcot your first foot problem?
- How long have you been receiving treatment for the Charcot for?
- What thoughts went through your mind when you were told you had been diagnosed with Charcot?
- What factors influenced your decision to agree to treatment with the cast/boot?
- Tell me about your experience of being treated for Charcot.
- How did you find being in a cast/boot?
  - Please can you tell me about any problems the cast or boot have caused you?
- Please can you describe how receiving treatment for Charcot has impacted on your day-to day life?
  - Are there things that you would like to do that you cannot because of the Charcot? Can you tell me about these?
  - Can you describe the impact of not being able to do the things you would like to do?
- How do you think receiving treatment for Charcot has impacted on your family/friends?
  - Can you explain in more detail what the impact has been?
  - Has anything happened as a result of this, can you tell me about it?
- Please can you tell me about what you think the long-term implications of CN will be for you and/or your family?
  - Can I ask you to describe how you think Charcot will affect you in the future?
