# Supplementary material for: A qualitative study to understand people’s experiences of living with Charcot neuroarthropathy
Source: Diabet Med. 2022 Jan 14;39(6):e14784. doi: 10.1111/dme.14784 (PMC9305882; doi:10.1111/dme.14784)
Supplement: Supplementary file 2 — Data S2 [file DME-39-0-s001.docx]

Supplementary material 2 - Codebook

| Name | Ref | Illustrative quote |
| --- | --- | --- |
| *Blame for developing CN attributed to themselves and healthcare professionals*. | | |
| Blame | 14 | I knew after the first Charcot in the left foot that I had to be more careful. And I suppose I blamed myself for not taking more care, when my husband was saying, come on, turn round we can go home. ‘No, I can walk another…’ |
| Fears and worries | 19 | Every time I get pain in this foot now, I’m thinking, oh my god. |
| Lack of awareness & raising awareness | 13 | I think the most important thing is education for people nobody knows about it not even people with diabetes on insulin, they don’t know, and they should |
| People’s perception what caused CN | 24 | I was given the boots, but I wasn’t given the information that I should ween myself off out of the air cast, so I didn’t. |
| Perceived benefits | 3 | But I have got my sugar levels down. |
| Problems following advice to rest | 18 | I’ve got to be very careful about the extra walking. If it isn’t necessary, I don’t do it; there are certain things I’ve got to do. |
| Problems getting a diagnosis | 52 | I think I had a fall and I kept going to the…I went to the hospital; to A&E and they just said it was a sprain, to carry on as normal. So, I kept going to work |
| Thoughts about diagnosis | 31 | They decided it was a CHARCOT foot and was told that I would be in plaster for a year. I thought I would kill them [Laughs], but I didn’t. |
| Thought’s future | 45 | Every time I get pain in this foot now, I’m thinking, oh my god. |
| *Disruption to people’s roles, responsibilities, relationships, and mobility, which people adapt to try address and manage.* | | |
| Adaptions to make life easier | 14 | I’ve had to do my main shopping online via computer instead of going out and looking. |
| Appearance | 17 | Just how it looks, makes me look like I’ve got a stigma, I’ve got a disability and I can’t do things normally. |
| Boredom | 9 | I’ll get fed up with that and I’ll read a magazine or something or I’ll…then I’ll get fed up with that and I’ll think, what can I do next? |
| Caring responsibilities for others | 38 | So that was me main worry; not about me, about me mum you see. So, I have got to function so that I can look after her, you know. |
| Difficulty doing daily household jobs (cleaning etc) | 32 | It’s just not being able to do anything, it’s horrible. I mean, I love cooking, but I can’t do it; I can’t cook standing up and then you’ve got sticks on – I can't take a hot saucepan off the stove |
| Emotional impact | 52 | I said to my husband, I could just sit down and sob me heart out but I’m not going to |
| Feelings about consequences work | 40 | I can’t do nothing; can’t obviously…can’t do stairs or anything. Um, I’ve had to finish my job because it involved all walking and |
| Guilt | 13 | You see then I feel under pressure because I’m putting pressure on her as well. So, that makes me feel awkward. |
| Help and support | 30 | Everyone is very kind and want to offer to do things when you can’t do them; I’ve had people do my shopping and that’s very kind of them. |
| How fill time | 11 | I go in the shed and count screws. [Laughs] You know, sort out nuts, screws and bolts. Sometimes I get frustrated. |
| Impact of CN on personal daily activities (washing etc) | 14 | Sometimes, she’s had to help me out of the shower because me balance isn’t very good. I’ve fell out the bath a couple of times, |
| Making yourself useful | 9 | I like it, if she gives me something to do where I need to find this – go on the internet and find this… |
| Mobility impact and adaptations | 85 | It made me not want to walk around. I did get some crutches but then I hurt my thumb on the crutches, so I had to stop using those. |
| Money worries | 29 | That’s another thing you’ve got to think about. The cost of everything. |
| Relationships communications with partners and spouses | 49 | It’s just horrible. I’m lucky I had a good one at the side of me, otherwise…[whispers – I don’t know what I’d have done.] |
| Talking about wearing casts & boots | 61 | It was such a bad experience for me at first, I was offered this heavy wellington boot its not suitable for me someone in their sixties. It needed pumping up manually it felt like a slab on my foot. |
| Transportation issues | 30 | I come in a taxi; it’s the only way I can get here, otherwise it’s three buses and I can’t do buses because I cant get up the steps. |
| Unsteadiness falls | 46 | I have had moments where I have crashed to the floor |
| Weight issues | 13 | I’ve put a stone on in weight after a year, which I’m not happy about. [Laughs] And now I’ve got back problems, due to all the sitting around. |
| What other people see and say | 21 | They talk to X. ‘What’s she done? Is she alright? Is she in pain?’ |
| *Pain which participants related to the direct or indirect consequences of wearing the cast or boot.* | | |
| Cast pain | 6 | When I went back to the clinic, they asked me were the boot was. I told them it crippled me, it was enough to finish me off |
| Foot pain | 12 | There’s pain with it that…when you’re a diabetic you’re not supposed to have any pain with it but there is |
| Nerve pain | 6 |  |
| No pain | 4 | So…it’s fine, it doesn’t hurt so there isn’t any discomfort or anything, |
| Other pain | 1 | I’ve got Raynaud’s as well and medication for Raynaud’s is no longer available. |
| Pain knees, hips etc | 7 | The most pain I get is from this foot and my hips, you know, from it being off balance. |
| Treatment for pain | 10 | It’s just so painful though and it’s the medication that needs sorting to get the combination right |
| *Trapped at home isolated and missing social life and daily life routines.* | | |
| Bereavement | 5 | We’ve just lost mum six-weeks ago, so, she was a big help, want she, she used to do a lot of stuff. I think we’ll manage; we’ve got no choice, we’ll have to. |
| Boredom | 9 | Im bored of watching tele until you…[laughs] there’s only so much daytime tv you can take. |
| Christmas | 7 | Couldn’t help her at Christmas but she has, she’s been a brick. I’ve aid it before but if it wasn’t for the wife…I don’t know. But luckily, I’ve got the old girl |
| Comparing their life to others worse off | 11 | Sitting in that waiting room, it ain’t very nice you know |
| Doing things with children | 1 | Yeah, I’m afraid they’re a bit like me, they’re geeks. So, there’s a certain amount of commonality there. |
| Emotional impact | 52 | Terrible, terrible, honestly. I wouldn’t wish this on me best mate or me worst enemy. |
| Exercising | 12 | I’m used to using the exercise to help the sugar. So, when I found that I couldn’t exercise as much, I started to think right, well, I won’t bother with that then, I’ll just trim the diet a bit more and try to balance it that way. |
| Families and not being able to visit | 29 | We used to visit family in X, but we can’t…we haven’t done that for a long time and that really hurts. |
| Going out and about | 54 | I’m used to being with people all the time, conversing, but I can’t now do that as much. I wish I could go out more |
| How life has changed - impact | 47 | Now, if I knew this was what would happen…pfft. It’s horrible, but I can see a bit of light at the end of the tunnel, so… |
| Loneliness | 8 | For the first seven months, apart from coming here, I was house bound. |
| Money worries (stops going out) | 29 | I’s the cost; it’s the cost of going out anywhere |
| Relationships communications with partners and spouses | 49 | It’s not fair on her because if we do go out, she can’t drink because obviously she’s driving and it does put a lot of the pressure on her, so…yeah |
| Things miss or can't do | 53 | Really, holidays are out of the question till I’ve been able to…everything’s going well with that. Um |
| Walking the dog and pets | 13 | I’ve had problems health wise but not being able to go out, not being able to take me dog for a walk. |
